# Factors Related to the Dignity of Older People in Tehran in 2020

**DOI:** 10.1093/geroni/igaa057.643

**Published:** 2020-12-16

**Authors:** Ali Darvishpoor Kakhki, Fereshteh Moradoghli, Roghayeh Esmaeili, Ali Kakhki

**Affiliations:** Shahid Beheshti University of Medical Sciences, Tehran, Iran

## Abstract

The elderly are the fastest growing segment of the population globally. This is also the case in developing countries such as Iran. The fear of aging and the refusal to accept older adults into the mainstream of society affects the dignity of older people. This study was conducted to describe factors and dignity of older people in Tehran, Iran. This descriptive, correlational study was conducted on a sample of 215 older people above age 60 in 10 public parks in five regions of Tehran in 2020. A socio-demographic questionnaire and patient dignity inventory were used for data collection. Content validity and Cronbach’s alpha were used for evaluating the validity and reliability of questionnaires. Data were analyzed with SPSS. 60% of subjects were male and 40% female with a mean age of 68(± 5.05) year. The mean scores for dignity domains ranged from 67.30 (±17.56) for symptom distress, to 93.01(± 10.90) for dependency on a 100-point scale. The scores of dignity domains were significantly associated with job status, housing status, income source, health insurance, chronic diseases, annual physician referral rates, and hospitalization in last year. The best predictors of dignity were health insurance and annual physician referral rates. The findings of this study showed that the dignity of older people is related to a number of factors. Monitoring modifiable factors such as health insurance and annual physician referral rates and non-modifiable factors such as chronic diseases will help us to preserve or improve the dignity of older people.

